# Supplementary material for: Characterization of Three Types of Elongases from Different Fungi and Site-Directed Mutagenesis
Source: J Fungi (Basel). 2024 Feb 3;10(2):129. doi: 10.3390/jof10020129 (PMC10890106; doi:10.3390/jof10020129)
Supplement: Supplementary file 1 [file jof-10-00129-s001.zip › jof-2845633-supplementary.pdf]

Table S1. Primers used in the present study.

**Primers**	**Sequence（5'-3'）**	**Purpose**
T7	TAATACGACTCACTATAGGG	PCR verification
CYC1 Terminator	TCGGTTAGAGCGGATGTG	PCR verification
McELO-F	CGGGGTACCTATGGATGTGGATAAGCTTCAACCT	Amplification
McELO-R	GCTCTAGACTACTCGGTCTTCTTGGCCTTACG	Amplification
PrELO-F	AAGCTTACCATGGGGGGTTCTCATCATCA	Amplification
PrELO-R	TCTAGATTACTGCGTCTTTTTCTTGTTCGACTTG	Amplification
PsELO-F	GAATTCATGGAGACCACCTTCGCCC	Amplification
PsELO-R	TCTAGATTACTGCGTCTTCTTGGCGACG	Amplification
Mc Q154A-F	TTCTCTCTTGGCTACCTATCATCACTCT	Mutagenesis
Mc Q154A-R	AGAGTGATGATAGGTAGCCAAGAGAGAA	Mutagenesis
Pr Y90A-F	TCCTCTGCTCGGCCATGTGCATGGA	Mutagenesis
Pr Y90A-R	TCCATGCACATGGCCGAGCAGAGGA	Mutagenesis
Pr M91A-F	CTCTGCTCGTACGCTTGCATGGAGG	Mutagenesis
Pr M91A-R	CCTCCATGCAAGCGTACGAGCAGAG	Mutagenesis
Pr Y104A-F	CTACCGCAACGGGCCTCGGCCACC	Mutagenesis
Pr Y104A-R	GGTGGCCGAGGCGCCGTTGCGGTAG	Mutagenesis
Ps L165A-F	CACCGTCCTCGCCTTCTGCTGG	Mutagenesis
Ps L165A-R	CCAGCAGAAGGCGAGGACGGTG	Mutagenesis
Ps Y256A-F	GTACTCGTCGGCCTTCGCCCTCTTC	Mutagenesis
Ps Y256A-R	GAAGAGGGCGAAGGCCGACGAGTAC	Mutagenesis

Table S2. Conversion of Polyunsaturated Fatty Acids by McELO, PrELO, or PsELO. Values are represented as the averages of percent conversions, [(% of generated fatty acid)/ (% of generated fatty acid+% of substrate fatty acid)] × 100, from three independent experiments.

Fatty acid substrate	% Conversion		
	PY-McELO	PY-PrELO	PY-PsELO
$C18:2^{\Delta9,12}$	8.4	1.9	0.0
$C18:3^{\Delta9,12,15}$	3.2	0.0	0.0
$C18:3^{\Delta6,9,12}$	0.0	74.3	0.0
C20:0	0.0	0.0	26.3
$C20:4^{\Delta5,8,11,14}$	0.0	0.0	0.0
$C20:5^{\Delta5,8,11,14,17}$	0.0	0.0	0.0

Table S3. Comparison of conversion rates of different sources of Δ6 elongase

△6-ELO origin	GLA additive quantity/μM	conversion rate /%	References
Green Microalga Parietochloris incisa	250	16.4	[1]
Mortierella alpina	25	60	[2]
Marine Alga Nannochloropsis oceanica	250	70.5	[3]
Physcomitrella patens	500	41	[4]
Conidiobolus obscurus	250	50	[5]
Thraustochytrium sp. ATCC 26185	100	64.3	[6]
Pythium sp. BCC53698	50	29.3	[7]
Myrmecia incisa Reisigl	/	23.89	[8]

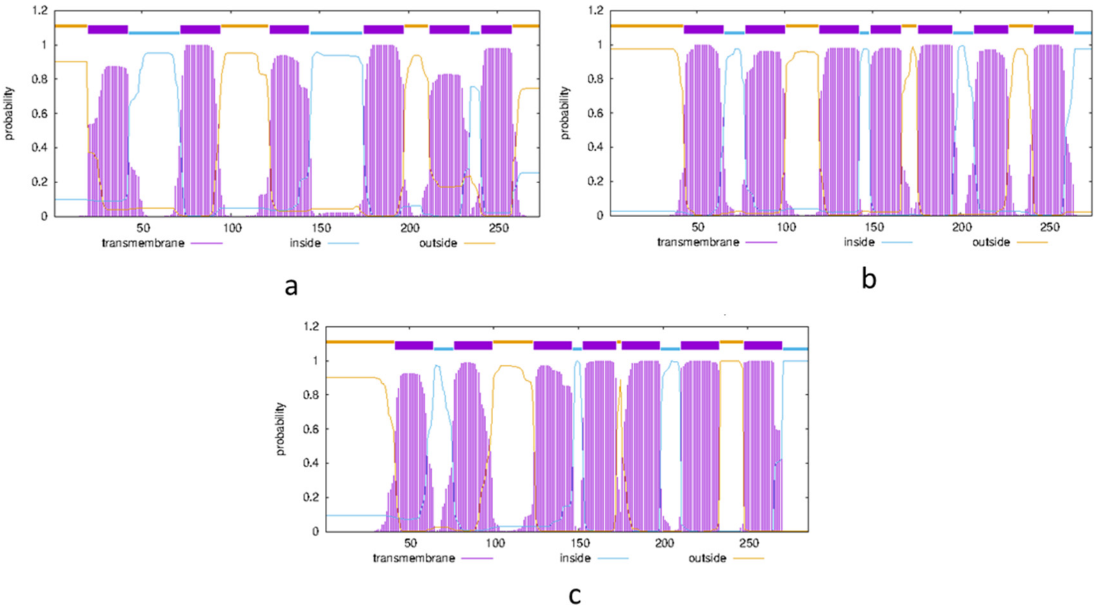

Figure S1. Predicated transmembrane domains for (a) McELO, (b) PrELO, (c) PsELO by TMHMM (V2.0, http://www.cbs.dtu.dk/services/TMHMM/).

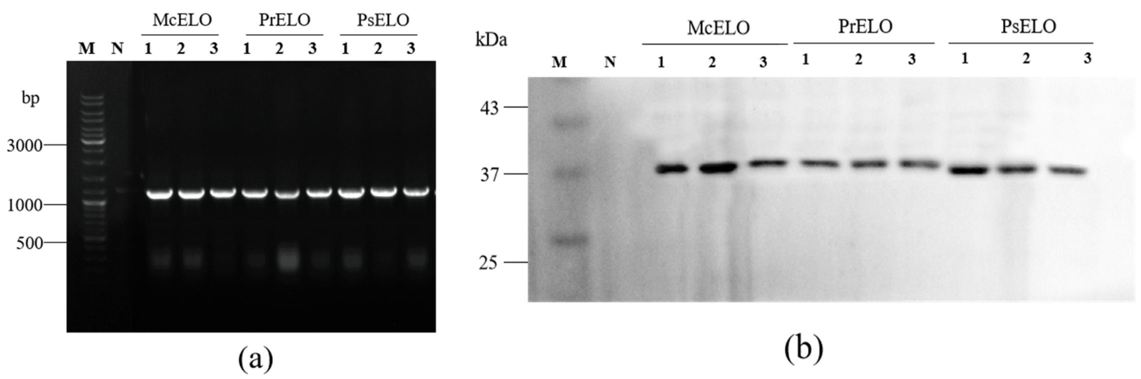

(a) (b)

Figure S2. Validation of *S. cerevisiae* transformants harboring McELO, PrELO, PsELO genes. (a) Validation of the recombinant strains *Escherichia coli*-pYES2- McELO, PrELO, PsELO; (b) Western blot analysis of pY-McELO, pY-PrELO, pY-PsELO. M: marker; N: negative control;

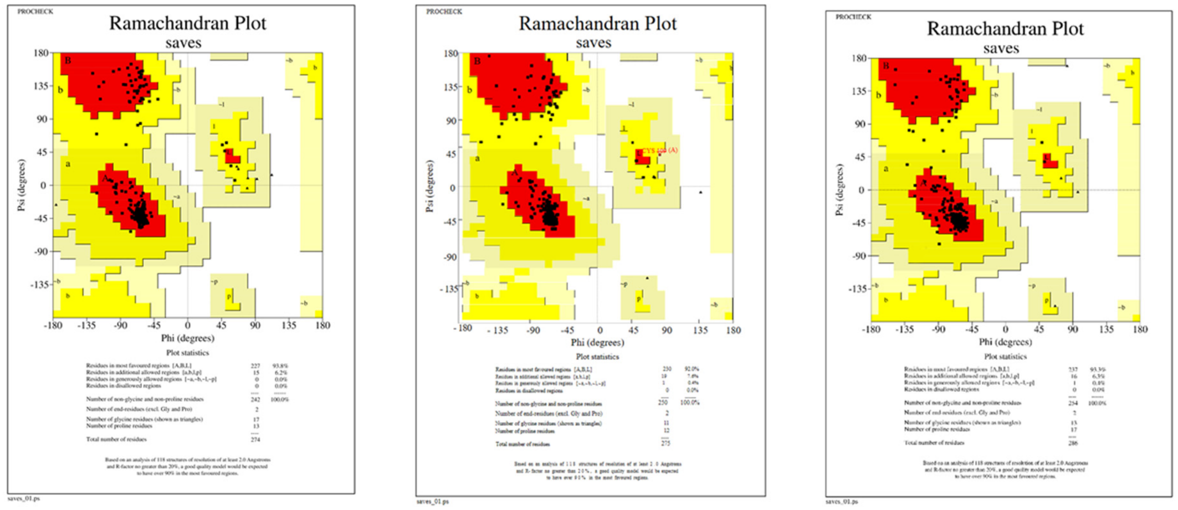

McELO PrELO PsELO

Figure S3. Ramachandran plot of a polypeptide backbone torsion angles psi (w) against phi (u) of amino acid present in the modeled structures of McELO, PrELO, PsELO generated by AlphaFold. The most favored regions are colored in red. The additional allowed regions [a, b, l, p] are colored in yellow. The generously allowed regions [~a, ~b,~l, ~p] are colored in pale yellow. All non-glycine and proline residues are illustrated as filled black squares, and glycine

(non-end) is indicated as filled black triangles. Disallowed residues are colored in white.

Reference:

1. Sakuradani, E.; Nojiri, M.; Suzuki, H.; Shimizu, S. Identification of a novel fatty acid elongase with a wide substrate specificity from arachidonic acid-producing fungus Mortierella alpina 1S-4. *Appl Microbiol Biotechnol* **2009**, *84*, 709-716, doi:10.1007/s00253-009-1999-3.
2. Jennifer M. Parker-Barnes; Tapas Das; Emil Bobik, A.E.L., Jennifer M. Thurmond, Lu-Te Chaung,; Yung-Sheng Huang, a.P.M. Identification and characterization of an enzyme involved in the elongation of n-6 and n-3 polyunsaturated fatty acids. *PNAS* **2000**, *97*, 8284–8289.
3. Shi, Y.; Liu, M.; Pan, Y.; Hu, H.; Liu, J. Delta6 Fatty Acid Elongase is Involved in Eicosapentaenoic Acid Biosynthesis Via the omega6 Pathway in the Marine Alga Nannochloropsis oceanica. *J Agr Food Chem* **2021**, *69*, 9837-9848, doi:10.1021/acs.jafc.1c04192.
4. Zank, T.K.; Za, U.; Beckmann, È.h.C. Cloning and functional characterisation of an enzyme involved in the elongation of D6-polyunsaturated fatty acids from the moss Physcomitrella patens. *The Plant Journal* **2002**, *31*, 255-268.
5. Tan, L.; Meesapyodsuk, D.; Qiu, X. Molecular analysis of Δ6 desaturase and Δ6 elongase from Conidiobolus obscurus in the biosynthesis of eicosatetraenoic acid, a omega3 fatty acid with nutraceutical potentials. *Appl Microbiol Biotechnol* **2011**, *90*, 591-601, doi:10.1007/s00253-010-3060-y.
6. Ohara, J.; Sakaguchi, K.; Okita, Y.; Okino, N.; Ito, M. Two fatty acid elongases possessing C18-Delta6/C18-Delta9/C20-Delta5 or C16-Delta9 elongase activity in Thraustochytrium sp. ATCC 26185. *Marine Biotechnology* **2013**, *15*, 476-486, doi:10.1007/s10126-013-9496-1.
7. Jeennor, S.; Cheawchanlertfa, P.; Suttiwattanakul, S.; Panchanawaporn, S.; Chutrakul, C.; Laoteng, K. Novel elongase of Pythium sp. with high specificity on Delta(6)-18C desaturated fatty acids. *Biochem Bioph Res Co* **2014**, *450*, 507-512, doi:10.1016/j.bbrc.2014.06.004.
8. Yu, S.Y.; Li, H.; Tong, M.; Ouyang, L.L.; Zhou, Z.G. Identification of a Delta6 fatty acid elongase gene for arachidonic acid biosynthesis localized to the endoplasmic reticulum in the green microalga Myrmecia incisa Reisigl. *Gene* **2012**, *493*, 219-227, doi:10.1016/j.gene.2011.11.053.
